# Systematic Review of Platelet-Rich Plasma Use in Androgenetic Alopecia Compared with Minoxidil^®^, Finasteride^®^, and Adult Stem Cell-Based Therapy

**DOI:** 10.3390/ijms21082702

**Published:** 2020-04-13

**Authors:** Pietro Gentile, Simone Garcovich

**Affiliations:** 1 Department of Surgical Science, Plastic and Reconstructive Surgery, “Tor Vergata” University, 00133 Rome, Italy; 2 Institute of Dermatology, F. Policlinico Gemelli IRCSS, Università Cattolica del Sacro Cuore, 00168 Rome, Italy; simgarko@yahoo.it

**Keywords:** regenerative plastic surgery, regenerative medicine, androgenetic alopecia, AGA, hair growth, platelet-rich plasma, PRP, hair loss

## Abstract

The number of articles evaluating platelet-rich plasma (PRP) efficacy in androgenic alopecia (AGA) have exponentially increased during the last decade. A systematic review on this field was performed by assessing in the selected studies the local injections of PRP compared to any control for AGA. The protocol was developed in accordance with the Preferred Reporting for Items for Systematic Reviews and Meta-Analyses-Protocols (PRISMA-P) guidelines. A multistep search of the PubMed, MEDLINE, Embase, PreMEDLINE, Ebase, CINAHL, PsycINFO, Clinicaltrials.gov, Scopus database, and Cochrane databases was performed to identify studies on hair loss treatment with platelet-rich plasma. Of the 163 articles initially identified, 123 articles focusing on AGA were selected and, consequently, only 12 clinical trials were analyzed. The studies included had to match predetermined criteria according to the PICOS (patients, intervention, comparator, outcomes, and study design) approach. In total, 84% of the studies reported a positive effect of PRP for AGA treatment. Among them, 50% of the studies demonstrated a statistically significant improvement using objective measures and 34% of the studies showed hair density and hair thickness improvement, although no *p* values or statistical analysis was described. In total, 17% of the studies reported greater improvement in lower-grade AGA, while 8% noted increased improvement in higher-grade AGA. Only 17% of the studies reported that PRP was not effective in treating AGA. The information analyzed highlights the positive effects of PRP on AGA, without major side effects and thus it be may considered as a safe and effective alternative procedure to treat hair loss compared with Minoxidil^®^ and Finasteride^®^.

## 1. Introduction

The number of articles evaluating autologous platelet-rich plasma (PRP) efficacy in androgenic alopecia (AGA) have exponentially increased during the last decade (2009–2019).

Autologous activated PRP (AA-PRP) and autologous not-activated PRP (A-PRP) are considered standard routine for dermatologists and plastic surgeon experts in hair growth (HG). Preparation procedures are not standardized yet and regenerative mechanisms are still the object of study due to their various growth factors (GFs). The effects of the GFs contained in AA-PRP and A-PRP in HG have been reported [1,2,3,4].

In particular, the anti-apoptotic effect of A-PRP and AA-PRP has been suggested as one of the most important factors stimulating HG via the activation of the Bcl-2 protein (anti-apoptotic regulator) and Akt signaling, improving the survival of dermal papilla cells (DPCs) during the hair cycle (H-C) [1,2,3,4]. Additionally, the upregulation of fibroblast growth factor-7 (FGF-7)/b-catenin signaling pathways with A-PRP treatment has been suggested to stimulate HG by inducing hair follicle stem cell (HFSC) differentiation as well as prolonging the anagen phase of the HG cycle [1,2,3,4]. It also seems to stimulate the perifollicular vascular plexus via the increase of vascular endothelial growth factor (VEGF) and platelet-derived growth factor (PDGF) levels, which have angiogenic potential [1,2,3,4].

AGA is characterized by the miniaturization of follicles with a diminishment of the anagen phase, accompanied by an increase in the percentage of resting hair follicles (HFs) and the telogen phase, producing microscopic hairs [5]. Additionally, lymphocytes and mast cells were identified around the miniaturized follicles [6], in the bulge area [7].

In a scalp suffering from hair loss (HLs), HFSC numbers remain unaltered, though the number of more actively proliferating progenitor cells particularly diminishes [8]. This concept suggests that a bald scalp either does not have an activator or has an inhibitor of hair follicle (HF) growth.

The total number of articles published on PRP in HLs is considerable (163 articles were identified in PubMed). The results are very encouraging for the vast majority of the identified articles.

As previously described [1,2,3,4,7,8,9], there are several methods, kits, and procedures aimed at the preparation of PRP. The differences depend on the centrifugation’s time and *g*-force or revolutions used (revolutions per minute -RPM-), platelet’s amount, GFs, and chemokine release. These differences are increased by wide biological and temporal variation [5]. Consequently, it seems to be very difficult to select which methods, kits, and procedures for PRP are better [6] or which are more or less adequate for treating different types of HLs. The efficacy of autologous PRP in patients who suffer AGA is clear and it has also been reported several times by authors [1,2,3,4,7,8,9].

Autologous regenerative procedures are represented beyond the PRP, also from adult stem cell-based therapy (ASCs-BT) to HFSC injections. Most recently, many articles [1,2,10,11] showed the efficacy of autologous micro-grafts, containing unexpanded HFSCs, obtained by mechanical detachment and slow centrifugation of scalp micro fragments (2 mm) according to minimal manipulation rules, with promising results. HFSCs may be considered a cell population containing human hair follicle mesenchymal stem cells (HF-MSCs) and human hair follicle epithelial stem cells (HF-ESCs) [10]. In addition, HF-MSCs, obtained by the intra- and an extra-dermal portion of the scalp, are named human intra and extra dermal adipose tissue-derived hair follicle stem cells (HD-AFSCs) as previously described [11].

In the present systematic literature review, the effectiveness of an autologous regenerative therapy focused exclusively on PRP treatment for AGA was evaluated, with the conclusion that PRP was effective in promoting HG in most articles.

## 2. Methods

### 2.1. Institutional Guidelines

The human autologous PRP is considered an emocomponent for non-trasfusional use, and thus it is subject to regulations that define transfusion activities. In order to better understand the sense of the current European Rules, it is necessary to differentiate between emocomponents for topical or infiltrative use and those used in cell therapy, which involves complex techniques of bioprocessing of therapeutic cells.

Reference is made to Regulation n.1394/2007 of the European Parliament (EC) and by the reflection paper on the characterization of cutting edge treatment medicinal products draft concurred, 20 June 2014 European Medicines Agency (EMA)/Committee for Advanced therapies (CAT)/600280/2010 Rev 1, in which the autologous applications in one-step surgery, minimal manipulations, and omofunctional utilization are situations that do not require good manufacturing practices (GMPs) rules for processing, good clinical practices (GCPs) for the clinical application, and the ethical committee’s endorsement.

It is necessary to highlight the differences between the productions of emocomponents suitable for cell therapy compared with those directed to topical or infiltrative use. While the first require special handling procedures and details of product derivation, the second, where the effect is extrinsic and an amplification of the physiological function at the site of insertion, is much simpler, with the product being easily derived by simple physical means, but nonetheless, it is important to include it within the legislation while not limiting its use. All the rules have a common purpose: To guarantee the quality and safety of the procedures and the products of transfusion medicine. The European rules related to the use of PRP were represented both by Decree of 9 November 2007, n. 207, ‘Implementation of Decree 2005/61/EC in means of traceability of blood components intended for transfusion and the notification of adverse and severe reactions’, and by the Legislative Decree of 9 November 2007, n. 208, ‘Implementation of Directive 2005/62/EC relating to a quality system of blood’.

Currently, the PRP preparation must be performed respecting in Italy *“Law-Decree of the Blood, 2 November 2015”*, dispositions related to the quality and safety parameters of blood and emocomponents, in which all patients receive detailed oral and written information about the study, including the risks, benefits, and alternative therapies, and sign an informed consent form before any study procedures, according to trasfusional service.

In this law-decree, it is established that each PRP procedure must take place in a structure authorized by the reference blood transfusion service by means of a specific agreement. This Italian law-decree established:

- Quantity of platelets to be obtained (1 × 10^6^ µL ± 20%);
- Exclusion criteria (platelets disorders, thrombocytopenia, anti-aggregating therapy, bone marrow aplasia, uncompensated diabetes, sepsis, and cancer);
- Fields of application of the PRP only on the basis of available scientific evidence and guidelines of the national blood center;
- Methods of preparation of the PRP (kits and procedure);
- How to use the PRP (only topical or infiltrative);
- Quality and sterility checks on the sample obtained;
- Blood volume to withdrew (within 55 cc for each patient);
- The volume of A-PRP and AA-PRP to be obtained (depending on the extension of the AGA area);
- Labeling of each sample of PRP;
- Informed consent;
- Adverse reaction form; and
- Data processing module.

All the PRP procedures must be performed in accordance with the European rules and institutional guidelines, and they must be conducted following the principles outlined in the Declaration of Helsinki and internationally consented ethics in clinical research [12], performing a quality assessment based on the Strengthening the Reporting of Observational studies in Epidemiology (STROBE) checklist [13] and respecting the national laws representing in Italy by “Law-Decree of the Blood, 2 November 2015”.

For all physicians that want to use PRP in this field, the authors suggest strictly respecting the guidelines highlighted in the Italian decree, as it was reported to follow all the GCPs. This systematic review was the object of a research contract (R. D. 1467/2017) between the first author and the University of Rome “Tor Vergata”, Italy.

### 2.2. Search Strategy

Due to the growing interest in hair restoration, a number of investigations have been conducted to assess the efficacy of A-PRP and AA-PRP as a treatment modality for hair loss. A systematic review protocol was developed in accordance with the Preferred Reporting for Items for Systematic Reviews and Meta-Analyses-Protocols (PRISMA-P) guidelines. The search was conducted in accordance with the PRISMA guidelines and the Cochrane handbook. A multistep search of the PubMed, MEDLINE, Embase, PreMEDLINE, Ebase, CINAHL, PsycINFO, Clinicaltrials.gov, Scopus, and Cochrane databases was performed to identify studies on HLs treatment with PRP searching without a language or publishing-time restriction; 163 articles using the keyword “platelet-rich plasma hair loss” and, 155 articles using the keyword “platelet-rich plasma androgenetic alopecia” were identified. Of the 163 articles initially identified, 53 articles were reviews (including 19 systematic reviews and 9 meta-analyses), 70 articles were clinical studies in AGA (including 12 clinical trials -randomized placebo-controlled trial/randomized, double-blind, placebo- and active-controlled, half-head study/double-blind, placebo-controlled pilot study/blinded, randomized clinical ttrial-), 13 article were related to alopecia areata, 3 articles to cicatricial alopecia, 2 articles to lichen planopliaris, 2 articles were pre-clinical models (mouse and rat), 5 articles were in vitro studies, and 15 articles were identified as biased (not correct match with the key word used).

#### 2.2.1. Study Assessment

The aim of the present systematic review was to assess the selected studies comparing local injections of PRP compared to any control for AGA. Studies included in this article had to match predetermined criteria according to the PICOS (patients, intervention, comparator, outcomes, and study design) approach. Criteria for inclusion and exclusion are specified as following: P-Patients (-inclusion criteria- age 18–76 years, males who showed AGA or male pattern hair loss (MPHL) in stage I–V controlled by the Norwood–Hamilton classification scale and females with AGA or female pattern hair loss (FPHL) in stage I–III controlled by the Ludwig classification scale; and exclusion criteria: Other types of alopecia, alopecia areata, cicatricial alopecia, lichen planopliaris, patient with platelets disorders, thrombocytopenia, anti-aggregating therapy, use of pharmacological therapeutics targeting AGA as Finasteride^®^, similar drugs, and/or antiandrogens in the earlier year, bone marrow aplasia, uncompensated diabetes, sepsis, cancer, an MPHL in stages over VI degree, a FPHL in stages over III degree, use of topical medicines for AGA as lotions as Minoxidil^®^, prostaglandin analogs, retinoid, or corticosteroids in the earlier year); I-Intervention (inclusion criteria: Local application of autologous PRP; exclusion criteria: Combined use of PRP with other products); C-Comparator (inclusion criteria: Any type of control, internal, external and different product; exclusion criteria: Not applied); O-Outcomes (inclusion criteria: Hair count, hair density, hair thickness and hair color improvement; hair loss reduction; exclusion criteria: Not applied); S-Study design (inclusion criteria: Clinical trial, randomized placebo-controlled trial/randomized, double-blind, placebo- and active-controlled, half-head study/double-blind, placebo-controlled pilot study/blinded, randomized clinical trial; exclusion criteria: Reviews, expert opinion, comments, letter to editor, case report, preclinical model (animal studies), in vitro studies, articles identified as bias (not correct match with the key word used, group of study < 10 patients, shorter follow up than 3 months). No limitations were applied on ethnicity or method of PRP processing.

This systemic review, performed on the PICOS approach in which only randomized placebo-controlled trials/randomized, double-blind, placebo- and active-controlled, half-head study/double-blind, placebo-controlled pilot study/blinded, randomized clinical trials, focused on PRP in AGA were analyzed, is considered an EBM 1a level study according the Oxford Centre for Evidence-Based Medicine (OCEBM), March 2009 (https://www.cebm.net/2009/06/oxford-centre-evidence-based-medicine-levels-evidence-march-2009/).

#### 2.2.2. Study Selection

Only AGA articles were considered and, for this reason, a total of 40 articles related to alopecia areata (*n* = 13), cicatricial alopecia (*n* = 3), lichen planopliaris (*n* = 2), pre-clinical model (*n* = 2), in vitro (*n* = 5), and bias (*n* = 15) were excluded initially.

**Scheme 1:**
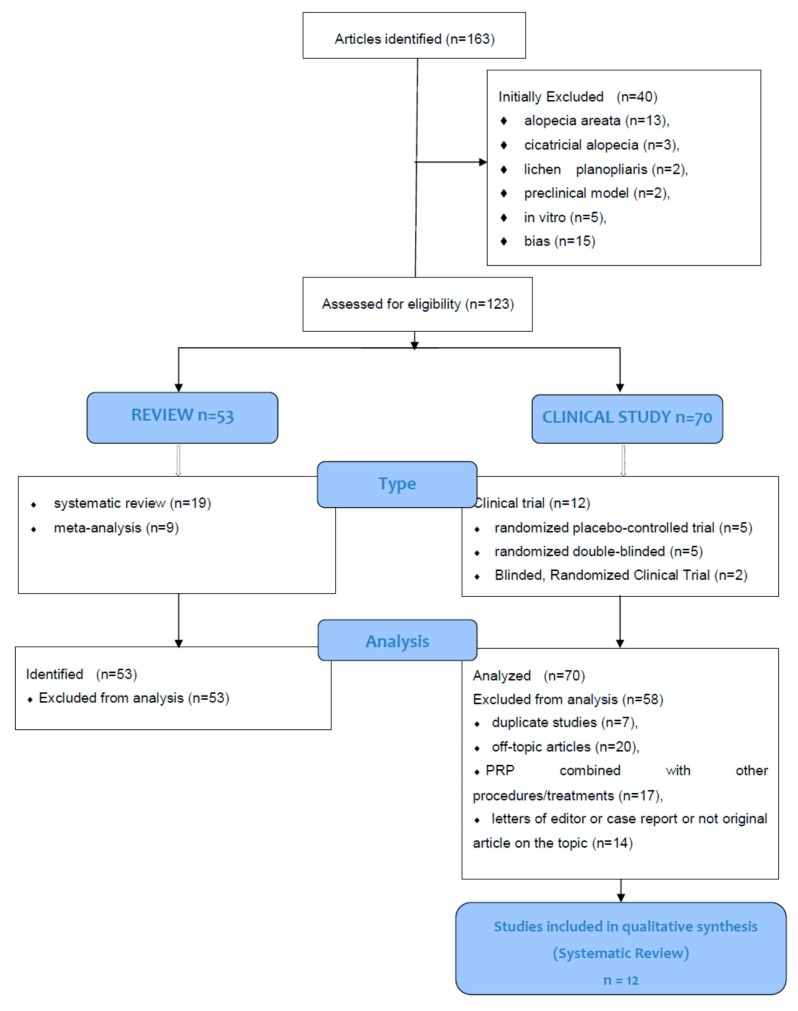
CONSORT (Consolidated Standards of Reporting Trials) flow diagram.

In total, 123 articles focused on AGA were identified and selected using Prisma Flow [14] (www.prisma-statement.org) (Scheme 1). Consequently, it was decided to include only clinical trials with male and female patients diagnosed with AGA, also referred to as MPHL or FPHL. In total, 53 articles were excluded as they were reviews, 7 articles were excluded as they were duplicate studies, 20 articles were excluded as they were off-topic, 17 articles were excluded as they assessed PRP in combination with other procedures/treatments, and 14 articles were excluded as commentaries or letters of the editor or case reports or not original articles on the topic. Twelve original studies were included in this systemic review. These 12 studies were evaluated and summarized by their study characteristics and study outcomes (Table 1), treatment protocols, and mode of PRP preparation (Table 2).

#### 2.2.3. Data Extraction

Data were independently collected by one investigator (PG) and checked by a second investigator (SG) only from the retrieved articles. Any disagreement on the collected data was settled by a consensus among PG and SG. No attempt was made to obtain specific or missing data from the authors. The following data were extracted: First author, year of publication, study design, number of patients, type of procedure, and primary and secondary outcomes.

The quality of the included studies was independently assessed using two investigators (PG and SG) using the Cochrane Collaboration’s Risk of Bias Assessment tool for RCT15 while using the Newcastle–Ottawa Scale to evaluate the individual non-randomized studies [25].

#### 2.2.4. Outcome Measures

The primary outcome was the difference in hair density (HD), number of hairs per cm^2^, and hair count (HC), number of hairs per 0.65 cm^2^. Secondary outcomes were hair thickness (HT) increase, hair re-growth (HRG), and hair cross-size (HCS) percentage increase.

All results collected from the studies were reported with the same measurements retrieved from the papers. From one paper, percentages were calculated from the patients’ individual data displayed in the paper [15]. The patient’s contralateral scalp was used as a control in some of the included papers, while in other studies, patients were respectively allocated into study groups when they underwent PRP and to the control group when they underwent the placebo or other treatments. Missing data were dealt with according to previously validated estimations [26,27].

### 2.3. Brief History Analysis of PRP Use in Androgenetic Alopecia

In total, 137 articles focusing on the use of PRP in AGA were published from 2015 to 2019 whereas only 18 were published before (range 2011–2014). Selecting original articles alone and excluding other types, Takikawa et al. [15] reported for the first time (2011) the effects of PRP-containing dalteparin and protamine microparticles (D/P MPs) on HG; Kang et al. [28] reported, in 2014, the clinical efficacy of interfollicular injection of a CD34+ cell-containing PRP preparation for patterned HLs; in the same year, Cervelli and Gentile [8] reported for the first time in the literature the clinical and histomorphometric evaluation of an AA-PRP injection in patients affected by AGA. In 2015, Gentile et al. [3] reported the most important (for the journal’s impact factor, actually SCTM 5,9 IF) randomized placebo-controlled clinical trial, including histological and trichospic analysis of the effect of AA-PRP and A-PRP in AGA.

Rodrigues et al. [29] reported recently (March 2019) the most important (for the journal’s impact factor, actually J Am Acad Dermatol 7,01 IF) double-blind controlled study, including platelet number and growth factor level analysis of the effect of PRP in AGA.

#### 2.3.1. A-PRP and AA-PRP Devices for Hair Regrowth

Seven different devices were clearly identified and analyzed. Currently, each of them could have a number of references that refer to commercial variants of a combination of fungible but that have a common denominator that is the device described. Used devices sorted alphabetically by trade name are as follows: Angel^®^ (Arthrex, Inc. Corporate Naples, Florida, 1370), Cascade^®^ (Musculoskeletal Transplant Foundation, Edison, NJ 08837) and Selphyl^®^ (Factor Medical, LLC Langhorne, PA 19047), C-Punt^®^ (Biomed Device, MO, Italy, 41126), i-Stem^®^ Preparation System (i-Stem, Biostems, Co., LTD., Seoul, South Korea 138–843), MAG-18^®^ (DTS MG Co., Ltd., Seul, Korea #B108-147), MyCells^®^ (Kaylight Technologies Ltd., Holon, Israel), and Regenlab^®^ (En Budron b2, 1052 Le Mont-sur-Lausanne, Swiss).

The Angel^®^ device allows the selction of the degree of the platelet concentration in a wide range (3^x^ to 18^x^). It was used to prepare A-PRP (3 mL) from a large volume of peripheral blood (120 mL). The A-PRP was then combined with 5 mL of platelet-poor plasma (PPP) to produce 8 mL of A-PRP with a 5-fold increase in the platelet concentration over the whole blood. The A-PRP collected was then triggered via the addition of 10% (*v*/*v*) calcium gluconate to obtain AA-PRP [4].

Using the Cascade^®^ or Selphyl^®^ device, AA-PRP was prepared from a small volume of blood (18 mL) collected in two different tubes (9 mL each one) from a peripheral vein using sodium citrate (ACD) as an anticoagulant. The tubes were centrifuged at 1100× *g* for 10 min, with the final aim of obtaining a platelet pellet; later the suspension contained in the tubes was activated through the switch into two tubes containing CaCl^2+^ to induce platelet activation and exocytosis of the alpha granules [3,8].

C-Punt^®^ consists of a 60-mL syringe in which whole blood (55 mL) was collected from a peripheral vein using sodium citrate as an anticoagulant. The syringe was centrifuged at 1200 rpm for 10 min; later, the autologous platelet suspension PPP and PRP obtained, in an amount of 23 mL, was inserted in a platelet selector device, and at the end of the procedure, 9 mL of A-PRP was harvested [3,4,9].

Using an hourglass system, the i-Stem^®^ Preparation System, autologous blood (17.7 mL) was harvested by adding ACD as an anticoagulant (2.2 mL). After the first spin (centrifugation at 3000 rpm for 6 min), the PPP portion (1 mL) and RBCs (red blood cells) (2 mL) were removed and the suspension was re-centrifuged for the second time (3000 rpm for 3 min). At the end of the procedure, 15 mL of A-PRP were obtained [2].

Mag-18 PRP^®^ is a hourglass system in which 18 mL of whole blood and 1 mL of ACD were collected and centrifuged two times; the first time at 3000 rpm for 10 min and second time at 3400 rpm for 6 min. Then, 1.5 mL of A-PRP were obtained in the middle portion of the hourglass, indicated as a buffy-coat. It is very similar to the i-Stem^®^ Preparation System and may be considered the evolution protocol [2].

PRP Regen Blood Cell Therapy^®^ tubes were used to obtain A-PRP (15 mL, 5 mL per BCT tube) from whole blood (24 mL) taken from a peripheral vein using ACD. The top 2 mL of A-PRP from each tube was then discarded, giving 9 mL of A-PRP. Alternatively, a Kit RegenLab^®^ (Regen Lab SA, Le Mont-sur-Lausanne, Switzerland) was used to process 40 mL of venous peripheral blood. Blood was collected in five ATS (autologous thrombin serum) Regen^®^ tubes (8 mL each). All tubes were centrifuged at 1500× *g* for 15 min. After the centrifugation, PRP activated (AA-PRP) by autologous thrombin consolidated in the tube [4].

#### 2.3.2. PRP and Growth Factors Assessment

A-PRP and AA-PRP contain at least six major GFs, including basic fibroblast growth factor (b-FGF), epidermal growth factor (EGF), transforming growth factor-β (TGF-β), insulin-like growth factor-1 (IGF-1), PDGF, and VEGF, which are released after platelet activation [2]. Each one of these major GFs is involved in a specific bio-molecular activity during HRG. In this way, in fact, it is possible to identify different types of PRP preparations depending on their cell content and fibrin architecture as reported:

- Leukocyte-poor PRP (LP-PRP) or pure platelet-rich plasma (P-PRP). PRP without leukocytes and with a low-density fibrin network after activation;
- Leukocyte-PRP (L-PRP). PRP with leukocytes and a low-density fibrin network after activation (most frequent);
- Leukocyte-poor platelet-rich fibrin (LP-PRF) or pure platelet-rich fibrin (P-PRF). PRF without leukocytes and a high-density fibrin network.
- Leukocytes platelet-rich fibrin (L-PRF). PRF with leukocytes and a high-density fibrin network.

As highlighted, there are too many protocols for the preparation of A-PRP and/or AA-PRP depending on the different time and rpm used, the number of platelets, and the availability of GFs and chemokines. There is also wide biological (between patients) and temporal (day to day) variation [5]. So, it is difficult to assess which kit for PRP preparation is better and which is worse [6].

In each case, the GFs serve to promote angiogenesis, follicular cell proliferation, and initiation of cell division, thus having a fundamental role in HRG [1,2,3,4,9].

The list of GFs present in PRP and their suspected mechanism in the treatment of AGA is reported in Table 3.

#### 2.3.3. Protocol: Manual Versus Mechanical and Controlled Hair Injection of A-PRP and AA-PRP

In 7 studies (58%) extracted by 12 clinical trials, the scalp was separated into several regions (half-head placebo-controlled study) in which a targeted and controlled area were identified.

The A-PRP and AA-PRP injections were performed manually more frequently (75%; 9/12) versus the mechanical and controlled injection (17%; 2/12), as published in all the articles identified by Gentile et al. [3,8], while massage of PRP on the scalp was performed in one article only (8%) [23].

Currently, it is not possible to accept that the infiltration of PRP into the scalp is done in a totally empirical way through the use of one’s hands. In fact, in this case, it is not possible to perform a homogeneous and precise infiltration. The PRP injection, done by the hand of a plastic surgeon or a dermatologist, it not able to compete with the mechanical and controlled infiltration achieved by a mesotherapy gun equipped with software capable of scheduling the amount of PRP delivered for each cm^2^ (0.2 mL), the depth (5 mm), and inclination of the needle.

Gentile et al. [1,2,3,4,9] realized an inter-follicular infiltration (0.2 mL/cm^2^) to AGA-affected regions at a depth of 5 mm utilizing a mechanical and controlled injection via the Ultim Gun^®^ (Anti-Aging Medical Systems, Montrodat, France) outfitted with a 10-mL Luer lock syringe with 30-gauge needle, in three sessions spaced 30 days apart.

Regarding the number of sessions performed, different protocols have been proposed.

Rodrigues et al. [29] reported four subcutaneous injections of PRP. Hausauer et al. [30] described, in a prospective randomized single-blinded trial conducted on 40 moderate AGA patients, two different protocols based on sub-dermal PRP injections: Protocol 1, in which three monthly sessions with a booster 3 months later were performed or protocol 2 in which sessions every 3 months were done. At 6 months, both protocols produced statistically significant increases in hair count (*p* < 0.001). These improvements occurred more rapidly and were greater for patients who underwent protocol 1 (mean percent change: protocol 1, 29.6 ± 13.6 vs. protocol 2, 7.2 ± 10.4; *p* < 0.001).

Schiavone et al. [31] performed two injections, with a 3-month interval between the two interventions. Jha et al. [32] performed autologous platelet-rich plasma injections with micro-needling over a period of 3 months at 3-week intervals. Tawfik et al. [33] performed weekly for a maximum total of four PRP sessions in females affected by AGA. Mapar et al. [22] performed PRP injections in two sessions, 1 month apart.

Gentile et al. [1,2,3,4,9] reported very interesting results from performing three treatments that were administered to each patient at 30-day intervals. At the end of the three treatment cycles, the patients showed a mean HC increase of 33.6 hairs in 0.65 cm^2^ and a mean increase in total HD of 45.9 hairs per cm^2^ compared with baseline values (*p* < 0.001).

Takikawa et al. [15] performed five treatments of 3 mL of PRP and D/P MPs at 2- to 3-week intervals.

All details are reported in Table 1 and Table 2.

## 3. Results

### 3.1. Results Performing Literature Scans: PRP Studies with Hair Density and Hair Count Improvement

In 2011, Takikawa et al. [15] performed the first controlled clinical trial of PRP containing D/P MPs in 26 patients affected by frontal or parietal HLs. Solutions of either PRP with D/P MPs, or PRP and saline were infiltrated at sites of HLs (13 patients each), with controls being the opposing sides with equal HD. Twelve weeks later after five treatments, an increased mean HC was seen in both PRP- and PRP&D/P MP-treated regions relative to control sites. Additionally, significantly increased HCS was observed in both PRP- and PRP&D/P MP-treated areas relative to control areas. The patients treated reported, via their own subjective evaluation, HLs reduction, and greater hair texture. Microscopic evaluation of punch biopsies displayed a thickened epidermis, proliferation of collagen fibers and fibroblasts, and greater amount of blood vessels around HFs in sites treated with PRP. No infections, hematomas, or other severe side effects were observed, though patients referred to temporary pain at the infiltration areas.

Several observational works published in 2014 all concluded that PRP may be considered effective for male and female patients affected by AGA.

In particular, Schiavone et al. [16] (2014) performed an observational work in which 64 patients received two infiltrations of L-PRP mixed with plasmatic proteins 12 weeks apart. Six months later, HC and HT were visibly improved; an average of 40.6% of the patients treated reached at least a moderate level of improvement.

Gkini et al. [17] led a prospective cohort study with 20 patients. Three injections later, they displayed increased HD compared to baseline at 3, 6, and 12 months after PRP (*p* < 0.001), as well as improvements in HD and HT. In this report, milder forms of HLs represented by patients classified as II°–III° degree of the Norwood–Hamilton scale responded better to the PRP procedure than more advanced cases, producing more interesting results on the vellus. The researchers also suggested that the PRP treatment appeared to lead to increases in HT more than HC.

Khatu et al. [18] also performed a very small prospective cohort study to evaluate the effects of PRP in 11 patients. Four PRP injections later, nine patients reverted to having a negative hair pull test. HD and HC were improved and, in particular, HC was noted to be increased from 71 to 93.09 on average. Both Gkini et al. [17] and Khatu et al. [18] assessed patients’ satisfaction, finding an average score of 7.1 and 7.0 out of 10, respectively.

Gentile et al. [3] performed for the first time in the literature (2015) a randomized blinded half-head study evaluating the effects of an inter-follicular injection of PRP (0.1 mL/cm^2^) with 30-gauge needles, in selected scalp sites. In this work, 23 males suffering from AGA were treated by performing the PRP infiltrations (without local anesthesia) three times at intervals of 30 days, in which the scalp of each patient was divided into four sites: Frontal, parietal, vertex, and occipital. PRP injections were performed on half of the scalp, while the other side received saline as a placebo (Table 1). The article reported a statistically significant increase in mean HC, HD, and terminal HD after three months of the last PRP injection compared to saline.

Cervelli et al. [8] performed a similar study, finding equally interesting results. All outcomes displayed a statistically significant improvement as reported in Table 1. In both articles, the histological evaluation indicated that the epidermal thickness and density of follicles were both increased compared to baseline (*p* < 0.05) two weeks after the last PRP injection.

Additionally, immunohistochemistry revealed that the percentage of Ki67+ cells was also increased in both basal keratinocytes of the epidermis and hair follicle bulge cells at 2 weeks after the PRP procedure (*p* < 0.05 compared with baseline), suggesting an increase in keratinocyte proliferation. The researchers also observed an increase in the small blood vessel amount around the HFs (*p* < 0.05 at 2 weeks after PRP compared with baseline), confirming the concept that PRP stimulates angiogenesis via the discharge of vascular GFs.

Another randomized blinded half-head study was performed one year later (2016) by Alves and Grimalt [20], testing the PRP injections in 22 patients divided into two groups: Group a, treated with 3 mL of PRP on the right side of the scalp and 3 mL of placebo on the left; and group b, treated with the same two suspensions on opposite sides of the scalp. Three and six months later, interesting improvements in mean anagen hairs, mean telogen hairs, HD, and terminal HD in PRP-treated sites were reported when compared with baseline (*p* < 0.05). Mean total HD was the only measure found to be significantly increased in PRP versus placebo-treated sites (*p* < 0.05).

Singhal et al. [19] (2015) performed a controlled clinical trial to compare PRP with medical treatments in 20 patients, with 8 males and 2 females in each treatment group. HG was observed in 6 patients after just 7 days and in 4 patients after 15 days. Three months later, all evaluated parameters (Table 1) displayed superior results in PRP-treated patients than in the control group, although no statistical analysis was shown on the analyzed data. In comparison, the patients who underwent medical treatments displayed no improvement in the hair pull test or HG.

In an open-labeled pilot study performed by Gupta et al. [23] (2017) involving 30 male patients, in which each one received six PRP massage treatments after the scalp was first activated by micro-needling, interesting outcomes were reported. In fact, six months later, a significant increase in HT and HD was reported by a blinded evaluator, displaying an improvement of 30.2 ± 12.2% (Table 1). The procedure response was more significant in those with lower-grade HLs in terms of HT (*p* = 0.0446) and HD (*p* = 0.0196). Efficacy was more evident in patients who had a shorter duration of disease prior to therapy and patients without family HLs history, with an improvement in HT (*p* = 0.0485 and *p* = 0.0272, respectively) and HD (*p* = 0.0096 and *p* = 0.0114, respectively).

Anitua et al. [24] (2017) reported the results obtained after five injections of plasma rich in growth factors (PRGF) in 19 patients affected by AGA. One year after the treatment, the mean HD, HT, and terminal/vellus hair ratio displayed a statistically significant improvement (*p* < 0.05). Histological analysis displayed an improvement in the epidermal thickness, peri-follicular neo-angiogenesis, and terminal/miniaturized hair ratio, as well as decreased perivascular inflammatory infiltrates.

### 3.2. Results Performing Literature Scans: PRP Studies without Hair Density and Hair Count Improvement

Only two articles did not display a statistically significant improvement in the results assessed. These articles were published by Mapar et al. [22] and Puig et al. [21], respectively.

Puig et al. [21] performed a double-blind randomized placebo-controlled multicenter trial involving 26 patients with FPHL. Fifteen females were randomized to the PRP group (study group) and 11 to the placebo group (control group). Researchers marked a 4-cm^2^ area in the central part of the scalp, where hair was repeatedly evaluated during the work using the HairCheck^®^. Patients of the study group received one infiltration of either PRP or normal saline within 4 cm from this area at week 0 (Table 2). At week 26, no statistically significant difference was found between the study and control groups in terms of HC (Table 1). Patients of the study group did, however, report a subjective reduction of the HL rate, and an improvement of HT, and ease of hair styling, which none of the control group participants noted. This work was the only study published in which the patients received only one PRP or placebo treatment.

The second article was a prospective half-head comparative pilot study performed by Mapar et al. [22] on 17 male patients affected by AGA. Researchers performed PRP or normal saline infiltration (Table 2) during two sessions 1 month apart. Outcomes displayed a mean decrease in the number of terminal and vellus hairs six months after the PRP, which was assessed using only a magnifying glass. Consequently, the researchers found no statistically significant difference in the results obtained between the treated area and baseline.

### 3.3. Critical Assessment of Study Design

Among the articles reviewed, it is evident that a standardized and widely shared protocol for the use of PRP is lacking, as well as standardized evaluation procedures.

In particular, there is a lack of a widely shared consensus regarding the preparation procedure, eventual addition of activators (calcium chloride -CaCl- Ca2+; thrombin, calcium gluconate, etc.), g-force, RPM and timing of centrifuge to be used, platelet concentration that must be attained, volume of blood to be collected, and amount of PRP to be used. Three works, for example, reported the use of CaCl as an activator [18,19,20], while two works reported calcium gluconate [17,22], one work reported PRGF activator [24], and two other works reported Ca^2+^ [3,8]. Protocols also varied in the number of sessions, time interval between treatments, administration procedure, and follow-up period.

In terms of study design, they ranged from pilot studies to randomized blinded trials, and this variation has contributed to the difficulty in interpreting the results across the included studies. The studies were stratified considering not only the use of PRP alone or PRP combined with other treatments, but also sex, degree of HLs, sample size, randomization, and control groups, further obscuring PRP treatment results.

Of the articles reviewed, seven mentioned the use of a control group [3,8,15,19,20,21,22], and five conducted the study without it [16,17,18,23,24]. Five articles mentioned the randomization of patients into study or control groups [3,8,20,21,22], while three specifically mentioned that they did not randomize patients [17,18,19], potentially introducing bias. Although a major part of the articles included both male and female patients [15,16,17,19,20,24], others included only males [3,8,18,22,23] or only females [21]. Since male and female patterns of HLs have different manifestations and may have different mechanisms, it may be inappropriate to extrapolate the results to both sexes in studies examining only a single sex. Moreover, most of the investigations were compromised due to the small sample sizes. Most studies enrolled only 10–30 subjects [3,8,17,18,19,20,21,22,23,24], and the largest article examined 64 patients [34]. All articles had inclusion and exclusion criteria.

### 3.4. Side Effects

No major side effects, such as scarring, progressive worsening, or infections, were reported in the analyzed articles. Only mild headache, tolerable and temporary pain during the procedure, mild itching and desquamation, and transient edema were reported by some subjects after PRP injection.

### 3.5. Considerations

In total, 84% of the studies (10/12) displayed a positive effect of PRP for AGA treatment. Among them, 50% of the studies (6/12) displayed a statistically significant improvement in the objective measures (e.g., HD, HT) following treatment with PRP [3,8,15,17,20,24] and 34% of the studies (4/12) reported hair improvement with PRP, although no *p* values or statistical analysis were described [16,18,19,23]. In total, 17% of the studies (2/12) reported a greater improvement in lower-grade AGA [17,23], while one reported increased improvement in higher-grade AGA [16]. In total, 9% of the studies (only one), a study conducted by Mapar et al. [22], concluded that PRP was not effective in treating AGA via analysis of the terminal and vellus HC. However, in this study, only two treatments were performed, and outcomes were assessed using a magnifying glass only, which may not be the best method to use to measure the results. Further, neither an objective team evaluation or a subjective patient self-assessment were performed.

Another study, by Puig et al. [21], did not report a significant improvement in HC or the hair mass index after PRP treatment. In this work, however, only one treatment was performed and the PRP injected was not activated, thereby impeding its full therapeutic potential. Nevertheless, subjective improvement was reported by the patients as lower HLs and improved HT [21]. In the articles in which no statistical analysis was displayed [16,18,19,23], the investigators remarked positively on the subjective parameters on HG, volume, coverage, and mean HD. Overall, all the investigations in which a minimum of three PRP treatments were performed displayed an improvement in at least one objective measure.

Among the articles reviewed, it appears to be evident that a standardized protocol for PRP preparation (kits/procedures/methods) and PRP injection (mechanical and controlled vs. manual) is lacking, as well as standardized evaluation methods (trichoscan/phototricograms/magnifying glass). Without such standardized parameters, it appears to be more difficult to adequately assess the effectiveness of PRP between the different studies in AGA patients, in terms of HG improvement. Performing this analysis, as briefly introduced before, certain methodological differences were noted. Regarding the procedure of PRP preparation, a lack of consensus regarding the choice of preparation method, eventual addition of activators, timing of centrifuge, G-force and RPM used, platelet concentration, volume of blood collected, and amount of PRP injected was noted (Table 2 and Table 3). In total, 25% of the articles (3/12), for example, used CaCl as an activator [18,19,20], while 17% of the articles (2/12) used calcium gluconate [17,22], 9% of the articles (only one) used PRGF activator [24], and other 17% of the articles (2/12) used Ca2+ for PRP activation [3,8].

Treatment protocols also varied in the number of sessions, time interval between procedures, administration procedure, and follow-up period (Table 2 and Table 3). In terms of the study design, the articles analyzed ranged from pilot studies to randomized blinded trials.

## 4. Discussion

### 4.1. PRP Compared with Minoxidil^®^ and Finasteride^®^

AGA produces a yearly worldwide market income of US$4 billion and a growth rate of 1.8%, demonstrating a developing consumer market [35].

Current drugs indicated for AGA with approval from the U.S. Food and Drugs Administration (FDA) include Minoxidil^®^ and Finasteride^®^.

The Minoxidil^®^ (pyrimidine derivate) lotion 2% was the first drug to receive approval by the FDA for AGA treatment in males (1988) and in females (1991) [36,37]. Minoxidil^®^ lotion 5% received approval in 1997 for males who suffered AGA followed by approval of the 5% foam in 2006 [36,37]. Minoxidil^®^ prolongs the anagen and increases the HF diameter through activation of prostaglandin endoperoxide synthase-1, which increases the level of prostaglandin E2 [36]. Minoxidil^®^ increases the survival of DPCs by increasing the Bcl-2/Bax ratio and by activating ERK and Akt [37].

Finasteride^®^ is a type II 5-alpha-reductase-inhibitor, which decreases dihydrotestosterone (DHT) by about 65% in the serum, prostate, and scalp. It was registered in Europe in 1992 for the treatment of benign prostatic hyperplasia [38,39]. The drug was registered in the U.S. (1993) and Europe (1994) for the therapy of mild to moderate AGA in male patients [38,39]. Oral Finasteride^®^ also prolongs anagen, with a gradual improvement of HT [38]. Finasteride^®^ has been shown to reduce the pattern of hair loss associated with an increased expression of caspase and apoptosis inhibitors, stimulating HG [39,40].

Alternative procedures, based on autologous regenerative therapies and a minimally invasive approach, are represented, as introduced, by PRP (A-PRP and AA-PRP) and ASCs-BT as adipose-derived mesenchymal stem cells (AD-MSCs) and HFSCs [1,2,3,4,9,10,11]. A more invasive surgical approach is represented by hair transplants, which is indicated only for patients affected by aggressive conditions of AGA and complete HLs [1].

Adil A et al. [41] conducted a systematic review and meta-analysis of randomized controlled clinical trials indexed in PubMed, Embase, and Cochrane, and searched up to December 2016, with no lower limit on the year. They selected only randomized controlled trials, based on the U.S. Preventive Services Task Force quality assessment process, conducted separately for five groups of studies, in which they tested in male patients low-level laser therapy (LLL-T), 5% Minoxidil^®^, 2% Minoxidil^®^, 1 mg Finasteride^®^, and 2% Minoxidil^®^ in females [41]. All treatments were superior to the placebo (*p* < 0.0001) in the five meta-analyses, suggesting that Minoxidil^®^, Finasteride^®^, and LLL-T were effective at promoting HG in male patients who suffered AGA and that Minoxidil^®^ was effective in females with AGA.

To better compare the size of clinical outcomes obtained by the use of Minoxidil^®^ and Finasteride^®^ with those obtained by A-PRP, AA-PRP, and HFSCs, it is necessary to analyze the most recent results in HD and HC improvement obtained for these treatments. In detail, in a recent article by Gentile et al. [2], the HD improvement for A-PRP 23 weeks after the third injection (each injection was performed three times every 30 days) was 28 ± 2% hairs/cm^2^ compared with the placebo (saline solution). In the same article, an HD improvement for HFSCs treatment 23 weeks after the second injection (each injection was performed two times every 60 days) was 29 ± 5% hairs/cm^2^ compared with saline [2].

In a study by Van Nestle et al. [42], 212 males suffering AGA were randomized to receive Finasteride^®^ 1 mg daily or a placebo for 48 weeks. At baseline, the mean total and anagen HC in the Finasteride^®^ group were 200 and 124 hairs, respectively (% anagen = 62%), and the anagen to telogen ratio was 1.74 (geometric mean). In the placebo group, the respective values were 196 and 119 hairs (% anagen = 60%) and 1.57. At week 48, the Finasteride^®^ group had a net improvement (mean ± SE) compared with the placebo in total and anagen HC of 17.3 ± 2.5 hairs (8.3% ± 1.4%) and 27.0 ± 2.9 hairs (26% ± 3.1%), respectively (*p* < 0.001). Furthermore, treatment with Finasteride^®^ resulted in a net improvement in the anagen to telogen ratio of 47% (*p* < 0.001), supporting favorable results on hair quality that contribute to improvements in HG.

In a recent study of Bao L. et al. [43] on the use of Minoxidil^®^ 5%, the mean improvement in the total HD from baseline to 24 weeks was 18.8/cm^2^ in patients managed with a topical application and 38.3/cm^2^ in patients managed with electrodynamics micro-needling treatments plus topical 5% Minoxidil^®^ [43].

Regarding the expenses/effectiveness ratio, if on the one side, the drugs discussed appear to be effective in AGA patients, on another side, they cause a dependence promoted by the need to take daily Finasteride^®^ or to apply Minoxidil^®^ topically for a long period of time; however, this was not inferior to 12–24 months according to the evaluated studies [42,43].

On the other hand, the use of autologous treatments may free the patient from the daily routine, but the greater invasiveness of the procedures may lead to people having less compliance.

### 4.2. PRP Comparison with Autologous Adult Stem Cell-Based Therapy (ASCs-BT)

Adult stem cells can be harvested, prevalently, from two different kinds of tissues: Fat and scalp. Adipose tissue (AT) is a very interesting source of MSCs, having multi-lineage separation potential. AT may be collected using a minimally invasive procedure represented by liposuction. The AT must be identified as an effective alternative source of stem cells (SCs) with respect to bone marrow (BM) during intra-surgical ACB-T, both for cellular wealth and expansion potential. A few patients may have constrained AT or insufficient levels for autologous cell collection, but, given the high frequency of AD-MSCs (their amount is 100 to 300 times higher than in BM), a small AT amount may be considered adequate for SC collection and isolation [1]. AD-MSCs and stromal vascular fraction cells (SVFs) are essential for the activation of the scalp’s ESCs, thus releasing GFs.

VEGF drives HG and the improvement of HFs’ size by the stimulation of angiogenesis. PDGF maintains the anagen phase, while IGF-I controls the HG cycle and hair cells’ separation [1]. Their action is aimed at angiogenesis improvement and enhancement of the blood supply to DPCs. Likewise, they have immune-modulatory and immune-suppressive actions via the release of prostaglandin E2 (PGE2), leukemia-inhibiting factor (LIF), and kynurenine [1]. MSCs and SVFs have paracrine effects via TB4, EGR-1, SDF-1, and MCP-1, acting on human HF cells [1]. In fact, TB4 contributes to the SCs being triggered in HF, improving their relocation into the follicle and their separation. SDF-1 acts by triggering EGR-1, expanding the cell tropism toward the follicle and stimulating angiogenesis. The activity of MCP-1, despite being an inflammatory factor, has a demonstrated tissue regenerative impact [1].

Since AGA is characterized by an important inflammatory infiltrate, being responsible for the release of a variety of inflammatory cytokines [44], it is likely that the anti-inflammatory and immune-modulatory properties of PRP or dermal and progenitor stem cells may favor HRG [44,45,46].

Stoll et al. [47] hypothesized in a pre-clinical model that superficial mechanical skin trauma produced with a micro-needling device would induce long-term HRG at the treated areas. Five weeks after micro-needling, HRG started, followed by a reduction in hyperpigmentation of the affected skin. After 12 weeks, there was a 90% improvement in scalp coverage on areas that previously suffered from HLs. Twelve months after the treatment, coat conditions remained stable [47].

As reported in a clinical model review performed by Ferting et al. [48], micro-needling may be considered a minimally invasive dermatological procedure in which fine needles roll over the skin to puncture the stratum corneum. This therapy may induce collagen formation, neovascularization, and it may favor GF release in the treated sites. It has been used in a wide range of dermatologic conditions, including AGA and alopecia areata (AA) [48].

For scalp tissue, recently, in two interesting articles published by Gentile et al. [10,11], a minimal manipulation procedure was tested and developed to separate HFSCs, based on the centrifugation of the scalp’s micro fragments obtained by several biopsies (2 mm), without an expansion or cell culture. In this procedure, cell counting and identification of CD44+ HF-MSCs and the CD200+ HF-ESCs were performed.

In patients suffering AGA with important HLs, the HFSCs number remains unaltered; however, the quantity of the more effectively multiplying progenitor cells significantly decreases, as reported by Garza et al. [49].

The reconstitution and/or the regeneration of a complete HF from seeded cells in culture conditions is yet to be investigated in tissue engineering [50].

HFs are known to contain a well-characterized niche for grown-up SCs: The bulge, which contains epithelial and melanocytic SCs [51].

SCs in the bulge area, an obviously differentiated structure inside the lower permanent portion of HFs, may create the inter-follicular epidermis, HF structures, and sebaceous glands [52,53].

ESCs can likewise reconstitute in a simulated in vivo framework into a new HF [54,55].

Yu et al. [51] displayed that human HFs contain an SC population with the potential of separation, with the consequent possibility of their differentiation in neurons, smooth muscle cells, and melanocyte progenitors in the induction medium. The information analyzed and reported demonstrates that Oct4-positive cells are available in human skin, and the majority of them are located in the HFs in vivo. Oct4 has a place with the family of POU-domain transcription factors that are regularly communicated in pluripotent cells of the developing embryo and mediate pluripotency [56]. Each mature HF is a regenerating framework, which physiologically experiences cycles of growth (anagen), relapse (catagen), and rest (telogen) various times in a grown-up’s life [57]. In catagen, HF SCs are kept in the bulge. At this point, the resting follicle re-enters anagen (regeneration) when legitimate molecular signals are given. Amid late telogen to early anagen change, signals from the dermal papilla (DP) induce the triggering of quiescent SCs into the bulge [58].

Numerous paracrine factors are involved with this crosstalk at various H-C stages and some signaling pathways have been implicated [59,60,61]. In anagen, SCs in the bulge offer ascent to hair germs; at this point, the transient increasing cells in the grid of the new follicle proliferate quickly to frame another hair filament [62].

As a matter of fact, the authors feel the need to better know and investigate which stage requires action is important, with the aim of improving HRG and obtaining HF regeneration. The regeneration of HFs was likewise observed in patients [34] when dermal sheath tissue was used; moreover, this is useful for regeneration of the DP structure. After implantation, the whisker DP was equipped for the promotion of HF regeneration by holding the data to decide the hair fiber type and follicle size [63]. The grafting of dermal-inductive tissue was limited as it is impractical to produce more HFs than the one obtained from the donor tissues. To defeat this constraint, diverse methodologies and exploratory models utilizing fresh or cultured isolated cells from both dermal and dermal/epidermal origins were investigated. The vast majority of them included neonatal and embryonic murine cells.

Balañá ME et al. [50] realized a dermal-epidermal skin substitute by seeding an a-cellular dermal grid with cultured HF-ESCs and DPCs, both obtained from the adult human scalp. This construct was grafted into a full-thickness wound produced on nude mice skin. In 14 days, microscopical structures reminiscent of a wide range of HFs’ embryonic structures were observed in the grafted region. These structures displayed concentric cellular layers of human origin and expressed k6hf, the keratin present in epithelial cells. Despite the fact that the presence of completely mature HFs was not seen, these results demonstrated that both epithelial and dermal cultured cells from the adult human scalp in a dermal scaffold can create in vivo structures similar to HF’s embryonic/germ structures, thus resulting in an HG improvement and hair regeneration.

Kalabusheva et al. [64] combined post-natal human DPCs and skin epidermal keratinocytes (KCs) in a hanging drop culture to build a simulated HF germ. Blended HF germ-like structures showed the initiation of epithelial-mesenchymal collaboration, including Wnt pathway activation and the expression of follicular markers. In this article, the authors analyzed the impact of DP cell niche components, including soluble components and extracellular matrix (ECM) molecules, during the time spent on the organoid assembly and growth. Their outcomes showed that soluble components had little effect on HF germ generation and the Ki67+ cell score inside the organoids despite the fact that BMP6 and VD3 effectively maintained the DP character in the monolayer culture.

Talavera-Adame et al. [35] reported the bio-molecular pathway involved in cellular treatment. Specifically, Wnt/β-catenin signaling was displayed as being fundamental for the growth and upkeep of DPCs [65,66]. The increment of Wnt signaling in DPCs evidently must be considered one of the most important factors that enhances HG, as reported by Tsai et al. [65].

Festa et al. [67] detailed that adipocyte progenitor cells bolster the SCs niche and help drive the complex HG cycle. This follicular regenerative approach is fascinating and raises the likelihood that one can drive or reestablish the H-C in males and females who suffer from HLs by stimulating the niche with autologous fat improved with stromal cells.

Furthering this concept, Perez-Meza D et al. [68] described and reported the safety and tolerability of advanced fat tissue injection in the subcutaneous scalp in patients who suffer from hereditary alopecia. The outcomes obtained displayed that the stem cell-enriched fat grafting in the scalp may represent a promising elective way to deal with treating HLs in people.

Additionally, Fukuoka et al. [69] displayed a mean increment of 29 ± 4.1 hairs in male patients and 15.6 ± 4.2 hairs in females treated with fat-derived stem cell-conditioned medium infusion.

As reported, the PRP-based therapy must be compared with ASCs-BT. It appears to be necessary to perform this comparison not only in HRG but also in different regenerative fields, such as wound healing [70,71,72,73], that may present similar bio-molecular pathway aspects.

The authors’ goals are to elaborate on a cellular mechanism approach in order to regenerate and promote one’s own natural GF release.

The use of autologous A-PRP/AA-PRP, ASCs-BT, and biotechnology aims to promote HRG via their use in isolated suspensions or in combination. This stems from the necessity to move from “substitutive surgery”, represented by transplants (organs, skin, cartilage, bone, etc.), to “regenerative plastic surgery” with the regeneration of organs, tissues, and hairs induced via autologous GFs and cells.

### 4.3. Evidence-Based Medicine’s Impact of PRP in AGA Treatment

One of the problems most encountered in the scientific community is the level of consolidated evidence-based medicine (EBM) offered by PRP in the treatment of AGA and hair loss, compared with FDA-approved treatments like Minoxidil^®^ and Finasteride^®^.

Many institutional guidelines of several countries are based on the EBM impact of a procedure/drug. Regarding PRP in hair loss and AGA, as reported, 19 systematic reviews, 9 meta-analyses, and 12 clinical trials are indexed currently. The number is theoretically more than sufficient to demonstrate a consolidated EBM and related effectiveness of PRP’s use in AGA. Practically, several governments affirm the need for more clinical trials, systemic reviews, and meta-analyses to accept, definitely, consolidated EBM related to PRP in AGA. The rationale of the present study was to contribute a systemic review of randomized/controlled/clinical trials (identified as EBM level 1a study), on the knowledge in this field, consolidating the EBM of PRP use in AGA, and reporting the most updated information compared with the last systemic reviews, and adding an EBM 1a study to this topic.

Most recently, a systemic review was published by Hausauer and Humphrey [74] on the PRP effects in several hair loss kinds, analyzing the impact, limits, and advantages. Moreover, they described its role in soft-tissue remodeling and rejuvenation. In agreement with Hausauer and Humphrey’s works, and starting on this basis, the authors decided to contribute as these authors, with an EBM 1a study focused only on one theme: “PRP use in AGA”.

## 5. Conclusions

This systematic review suggests five fundamental points: First, the information analyzed highlights the positive impacts of A-PRP and AA-PRP on hair loss in AGA patients, as displayed by in vivo, in vitro, present a safe and effective alternative procedure to treat hair loss compared with Minoxidil^®^, Finasteride^®^, and Dutasteride^®^; third, it is necessary to perform three injections of PRP at least; fourth, PRP injections work better in male patients with low- or moderate-grade AGA; and fifth, PRP infiltration must be performed with mechanical and controlled injections.

The authors believe that the future will be based exclusively on regenerative-based therapies, and for this reason, invite all the audience to improve the level of publications in this field by focusing prevalently on EBM level 1–2 studies.

## Figures and Tables

**Table 1:** The study design and results of the included studies. Abbreviations: M, male; F, female; wks, weeks; mos, months. *p value not reported.

Authors	Study Type				Characteristics of Enrolled Subjects (Completed Study)	Objective Measures	Objective Assessment of Hair Growth	Subjective Assessment of Hair Growth	Year	Ref
	**Randomized**	**Controlled**	**Blinded**	**Half-head**						
Takikawa et al.	-	Yes	No	No	26 (26) 16 M, 10 F, aged 28–59, thin hair in the frontal or parietal areas	1. Mean number of hairs (digital and dermoscopic imaging) 2. Mean HCS of hairs (digital and dermoscopic imaging) 3. Epidermal thickness, collagen and blood vessel density around hair follicles (4-mm punch biopsy)	1. Yes* 2. Yes (*p* < 0.01) 3. Yes*	Patients reported less depilation when shampooing, greater bounce/resilience of hair, maintenance of healthy hairs	2011	[15]
Schiavone et al.	-	No	No	No	64 (64) 42 M, mean age 28, stage II–V; 22 F, mean age 32, Stage I–II	1. Hair count and hair thickness using Jaeschke 15-point scale rating of clinical change (macrophotographs examined by 2 independent evaluators)	1. Yes (mean change in clinical rating of 3.2 and 3.9)*	N.a	2014	[16]
Gkini et al.	No	No	No	No	22 (20) 18 M, aged 24–72, stage II-5a; 2 F, aged 58–72, Stage I	1. Hair pull test 2. Hair density and quality (dermoscopic photomicrographs and macroscopic photographs)	1. Yes* 2. Yes, *p* < 0.001; overall improvement in hair density and quality per photographs	Patient self-assessment questionnaire: mean result rating of 7.1 on a 1–10 scale; 85% reported improvement in hair quality and thickness; 65% reported increases in hair density	2014	[17]
Khatu et al.	No	No	No	No	11 (11) 11 M, aged 20–40, stage II–IV	1. Hair pull test 2. Hair count (Trichoscan) 3. Hair loss (clinical examination, macroscopic photos)	1. Yes (81.81% achieved a negative pull test at 12 wks.) 2. Yes (average mean gain of 22.09 follicular units/cm^2^)a 3. Yes (moderate improvement in hair volume and coverage with reduction in hair loss)	Patient satisfaction questionnaire: mean overall satisfaction rating of 7 out of 10	2014	[18]
Cervelli et al.	Yes	Yes	Yes	Yes	10 (10) 10 M, aged 20–52 stage IIa–IV	#1–4: Computerized phototrichogram and global photography: 1. Hair count 2. Hair density 3. Terminal hair density 4. Epidermal thickness and hair follicle density (3-mm punch biopsy) 5. Percentage of Ki67+ keratinocytes & blood vessel density (immunohistochemistry)	1. Yes (*p* < 0.0001) at 3 mos 2. Yes (*p* < 0.0001) at 3 mos 3. Yes (*p* = 0.0003) at 3 mos 4. Yes (*p* < 0.05) at 3 mos 5. Yes (*p* < 0.05) at 14 wks.	Physician and patient global assessment scale—results not reported	2014	[8]
Gentile et al.	Yes	Yes	Yes	Yes	23 (20) 20 M, aged 19–63 stage IIa–IV	#1–3: Computerized phototrichogram and global photography: 1. Hair count and total hair density 2. Terminal hair density 3. Epidermal thickness and hair follicle density (3-mm punch biopsy) 4. Keratinocyte proliferation and small blood vessel proliferation around hair follicles (immunohistochemistry) 5. Relapse of AGA	1. Yes (*p* < 0.0001) 2. Yes (*p* = 0.0003) 3. Yes (*p* < 0.05) 4. Yes (*p* < 0.05) 5. Four patients reported progressive hair loss at 12–16 mos	Physician and patient global assessment scale)—results not reported	2015	[3]
Singhal et al.	No	Yes	No	No	20 (20) 16 M, aged 25–32 4 F aged 32–35	1. Hair count (hair pull test) 2. Hair growth, hair volume, hair quality, fullness (global photographs)	1. Yes, pulled hair count was reduced by 65% (vs. 0% in controls)* 2. Yes, hair growth noted in 6 patients after 7 days but in 4 patients after 15 days; yet, all patients (10) had good hair growth after 3 mos*	N.a	2015	[19]
Alves and Grimalt	Yes	Yes	Yes	Yes	25 (24) 11 M, aged 18–65, stage II–V; 11 F, aged 18–86, Stage I–II	#1–6: Phototrichogram and global photography 1. Anagen hair (%) 2. Telogen hair (%) 3. Anagen: telogen ratio 4. Hair density 5. Terminal hair density 6. Hair count	PRP vs. placebo: 1–3, 5, 6. No (*p* > 0.05) 4. Yes, at 3 and 6 mos (*p* < 0.05) PRP vs. baseline: 1–5. Yes (*p* < 0.05) 6. No (*p* > 0.05)	N.a	2016	[20]
Puig et al.	Yes	Yes	Yes	No	26 (26) 26 F, stage II	1. Hair count (photography) 2. Hair mass index (Cohen HairCheck^®^ system	1. No (*p* = 0.503) 2. No (*p* = 0.220)	13.3% of treatment group vs. 0% of control group reported substantial improvement in hair loss, rate of hair loss, hair thickness, and ease of managing/styling hair; 26.7% of treatment group vs. 18.3% of control group reported feeling coarser/heavier hair	2016	[21]
Mapar et al.	Yes	Yes	Yes	Yes	19 (17) 17 M, aged 24–45, stage IV–VI	1. Terminal hair count (magnifying glass) 2. Vellus hair count (magnifying glass)	1. No (*p* = 0.25 at 6 mos) 2. No (*p* = 0.23 at 6 mos)	n.a.	2016	[22]
Gupta et al.	-	No	No	No	30 (30) 30 M, aged 25– 35 stage III–VII	1. Hair density (CapilliCare trichoscan) 2. Hair diameter (CapilliCare trichoscan) 3. Independent observer clinical evaluation (global macrophotographs)	1. Yes (39.7 ± 16.5% increase compared to baseline)* 2. Yes (39.8 ± 17.2% increase compared to baseline)* 3. Average improvement = 30.2 ± 12.2%	Patient self-assessment questionnaire: treatment group reported 30 ± 13.1% mean improvement (range 10–70%); 93.3% reported complete cessation of hair fall by 2 mos; 66.7% reported increase in hair growth; 36.7% reported improvement in hair texture	2017	[23]
Anitua et al.	No	No	Yes	No	19 (19) 12 M, aged 27–60, stage III–VI; 2 F, aged 32–60, stage II-frontal	#1–4 Computerized phototrichogram 1. Hair density 2. Hair diameter 3. Terminal/vellus-like hair ratio 4. Thin/regular/thick hair shafts among terminal follicles 5. Independent observer clinical evaluation (mean improvement score using global macro-photographs) 6. Epidermal thickness perivascular inflammatory infiltrate, rete ride number, terminal/miniaturized hair ratio, and collagen, reticular fiber and elastic fiber mesh quantity (3 mm punch biopsies) 7. Proliferative epidermal/follicular cells, newly formed blood vessels, and presence of bulge stem cell niches (immunohistochemistry)	1. Yes (*p* < 0.05) 2. Yes (*p* < 0.05) 3. Yes (*p* < 0.05) 4. Yes (*p* < 0.05) 5. Yes; 0.75/1* 6. Yes (*p* < 0.05 for most) 7. Yes (*p* < 0.05 for most)	Patient self-satisfaction score following a Likert scale: 7 = very satisfied, 6 = satisfied, 5 = indifferent, 1 = unsatisfied, and 0 = very unsatisfied; most patients (15/19) declared noticeable hair loss decrease, 13/19 declared noticeable improvement in hair quality and appearance, and 11/19 stated they would continue with PRGF treatment	2017	[24]

**Table 2:** Treatment protocols for the included trials. Abbreviations: n° treat, number of treatments; Int, interval; Centrif. Time, centrifugation time.

Authors	PRP n° Treat	Int	Max F-up	Type of PRP Injections	Protocol	Activators	RPM orG	Centrif. Time	Blood Volume	PRP Volume
Takikawa et al. [15]	5	2–3 wks	12 wks	Subcutaneous injection (3 mL) into selected 1 × 1 cm areas measured from the nasal tip and upper part of the auricular base	Manual Double Spi	-	a. 1700 rpm b. 3000 rpm	a. 15 min b. 5 min	15 mL	3 mL
Schiavone et al. [16]	2	3 mos	6 mos	After local anesthesia (xylocaine 1%, with adrenaline 1:100,000) was administered, cutaneous inflammation was induced via application of gentle pressure using 1.0-mm-deep Scalp-roller to favor activation of injected platelets; then, superficial injections were administered 1 cm apart	GPS III Platelet Separation System a. Single spin at baseline b. Double spin at 3 months	No (Scalp roller used to favor– platelet activation	-	-	a. 60 mL b. 40 mL	a. 6–8 mL PRP + 3–4 mL of plasmatic protein concentrate = 9–12 mL; 0.2–0.3 mL per injection b. Same as above
Gkini et al. [17]	3 (+1 booster)	21 days (booster 6 mos after onset)	1 year	Injections (0.05–0.1 mL/cm^2^) were performed using nappage technique in affected areas to a depth of 1.5–2.5 mm; a specific area was checked at all times by defining a “V” (Kang’s point)	RegenA-PRPCentri (Regenlab) Single spin method	Calcium gluconate (0.1 mL per 0.9 mL of PRP; 1:9 ratio)	1500× *g*	5 min	16 mL	6 mL (0.05–0.1 mL/cm^2^)
Khatu et al. [18]	4	2 wks	12 wks	Nappage technique injections (2–3 mL) into a prefixed 1 × 1 cm squared area over the right parietal area; anesthetic cream was applied before each treatment after cleaning the skin with cetavlon, spirit, and povidoneiodine	Manual Double Spin	Calcium chloride (1:9 ratio)	a. 1500 rpm b. 2500 rpm	a. 6 min b. 15 min	20 mL	2–3 mL
Cervelli et al. [8]	3	4 wks	1 year (at baseline and 14 wks, 6 mos, and 12 mos after initial treatment)	Intradermal injections (0.1 mL/cm^2^) into 2 of the 4 selected halves (e.g., frontal or parietal) (placebo was injected into the other 2 halves) after the scalp was cleansed with 70% alcohol; local anesthesia was not used	Cascade-Selphyl-Esforax Kit	Ca^2+^	1100× *g*	10 min	18 mL	9 mL
Gentile et al. [3]	3	4 wks	2 years (at baseline and 2, 6, 12, 16, and 23 mos after initial treatment)	Interfollicular injections of PRP (0.1 mL/cm^2^) within 2 of the 4 selected areas of the scalp (physiologic solution into the other 2 areas), after cleaning skin with 70% alcohol; target areas were marked with semi-permanent tattoos for subsequent treatment and evaluation; local anesthesia was not used	a. Cascade-Selphyl- Esforax system b. PRL platelet-rich lipotransfert system c. C-punt system	a. Ca^2+^ b. Nothing c. Nothing	a. 1100× *g* b. 1200 rpm c 1200 rpm + double spin 1900 rpm	a. 10 min b. 10 min c. 10 min +10 min	a. 18 mL b. 55 mL c. 55 mL	a. 9 mL b. 20 mL c. 20 mL
Singhal et al. [19]	4	2–3 wks	3 mos (at 1-wk intervals)	Injections using nappage technique (multiple small injections in linear pattern 1 cm apart) after area was cleansed with spirit and povidone-iodine	Double spin method	Calcium chloride (9:1 ratio)	a. 1500 rpm b. 2500 rpm	a. 6 min b. 15 min	20 mL	8–12 mL
Alves and Grimalt [20]	3	4 wks	6 mos (at 3-mo intervals)	Injections (0.15 mL/cm^2^) within four 1 × 1 cm selected circular areas of the frontal and occipital scalp (marked with a dot tattoo) depending on the treatment-designated side of the scalp (vs. control side of the scalp received placebo (normal saline); no local anesthesia was used	Single spin method	Calcium chloride (10%, 0.15 mL)	460× *g*	8 min	18 mL	3 mL
Puig et al. [21]	1	N.a	26 wks (at 4-wk intervals)	Single subcutaneous injection within the 4 cm^2^ area in the central scalp (termed the “hair check data box”), after anesthesia (2% lidocaine and 0.5% bupivacaine) was administered	Angel PRP system (Cytomedix)	Nothing	-	-	60 mL	10 mL
Mapar et al. [22]	2	4 wks	6 mos (at 1, 3, and 6 mos after initial treatment)	Injections (1.5 mL of PRP) within one of two 2.5 × 2.5 cm square regions, at least 3 cm apart, in the scalp randomly assigned to be a case square (control square received 1.5 mL of normal saline); randomization of case and control squares was performed using a random number table; iron oxide- and titanium dioxide-containing substances were used to tattoo the corners of the squares	Double spin method using Tubex PRP tube (Moohan Enterprise)	Calcium gluconate (0.1 mL per mL of PRP)	a. 3000 rpm b. 3300 rpm	a. 6 min b. 3 min	9 mL	1.5 mL
Gupta et al. [23]	6	2 wks	6 mos	Scalp was activated by micro-needling; then, PRP was massaged into the vertex of the scalp (10 cm from the glabella)	Double spin method	-	-	-	-	-
Anitua et al. [24]	5	1 mo for first 4 sessions; final session 7 mos after start point	1 year	Intradermal injections of PRGF into hair-depleted areas	Single spin method	PRGF activator (BTI Biotechnology Institute)	580 rpm	8 min	18 mL	3–4 mL

**Table 3:** List of GFs identified in PRP and their suggested bio-molecular pathway in the treatment of AGA.

Growth Factors	Bio-Molecular Pathway in Hair Re-Growth
VEGF	Improves perifollicular angiogenesis; Elevated expression in dermal papilla cells during anagen phase; Endothelial cell-specific mitogen; Micro-vascular permeability and perifollicular vascularization;
EGF	Improves the activity and growth of follicle outer-root sheath cells by activation of Wnt/β-catenin signaling; Cell growth modulator during follicular differentiation; Proliferation and migration of follicular outer root sheath cells;
FGF	Improves the advancement of hair follicles; Anagen phase induction via B-catenin expression; Angiogenesis; Dermal fibroblast and hair follicle mitogen;
PDGF	Up-regulate the genes associated with HF separation, induction, and control of anagen; Angiogenesis and vascularization; Hair follicle dermal stem cell proliferation; Mesenchymal stem cell mitogen;
IGF-1	Improves the migration, survival, and proliferation of HF cells; Hair follicle proliferation during development; Increase hair density and inhibit apoptosis;
HGF	Enhance the proliferation of follicular epithelial cells Hair follicle elongation; Inhibits catagen phase induction;
TGF-ß	Stimulates the signaling pathways that manage the Hair cycle; Extracellular matrix synthesis; Fibroblast and mesenchymal stem cell proliferation; Hair folliculogenesis and maturation;
IL-6	Involved in WIHN through STAT3 enactment
IGFBP-1 to -6	Manages the IGF-1 effect and its connection with extracellular matrix proteins at the Hair follicle level
BMP	Maintains the DPC phenotype (fundamental for stimulation of HFSCs)
BMPR1	Maintains the proper identity of the DPCs (basic for explicit DPC work)
M-CSF	Involved in wound-induced hair growth
M-CSFR	Involved in wound-induced hair growth
Wnt3a	Involved in HF advancement through β-catenin signaling
PGE2	Stimulates anagen in HF
PGF2α	Enhance change from telogen to anagen
BIO	GSK-3 inhibitor
PGD2	Enhances follicle regeneration
Iron and l-lysine95	Still under examination

## Abbreviations

AGA	Androgenic Alopecia
PRP	Platelet-Rich Plasma
PPP	Platelet-poor plasma
A-PRP	Autologous-Non-activated Platelet-Rich Plasma
AA-PRP	Autologous-Activated Platelet-Rich-Plasma
HG	Hair Growth
HRG	Hair re-growth
GFs	Growth Factors
VEGF	Vascular endothelial Growth factors
PDGF	Platelet Derived Growth factors
IGF-1	Insulin like Growth factor-1
TGF-ß	TGF-ß: transforming growth factor-beta
EGF	Epidermal growth factor
PRGF	Plasma rich in growth factor
DPCs	Dermal papilla cells
DP	Dermal papilla
HD	Hair density
HC	Hair count
HT	Hair thickness
H-C	Hair cycle
HCS	Hair cross-size
EC	European Committee
CAT	Committee for Advanced treatments;
GMP	Good Manufacturing Practices
GCP	Good Clinical Practices
GFs	Growth factors
HF-MSCs	Human follicle mesenchymal stem cells
HFSC	Human follicle stem cells
HF-ESCs	Hair Follicle Epithelial Stem Cells
KCs	Skin epidermal keratinocytes
ECM	Extracellular matrix
HF	Hair Follicle
HFs	Hair Follicles
SVFs	Stromal vascular Fraction Cells
AD-MSCs	Adipose-derived Mesenchymal Stem Cells
SCs	Stem Cells
HLs	Hair Loss
HD-AFSCs	Human Intra and Extra Dermal Adipose Tissue-Derived Hair Follicle Stem Cells
RPM	Right per minute
MPHL	Male pattern hair loss
FPHL	Female pattern hair loss
ASCs-BT	Adult stem cells based therapy
AT	Adipose tissue
BM	Bone marrow
HA	Hyaluronic acid
DHT	Dihydrotestosterone
D/P	Dalteparin
D/P MPs	Dalteparin and protamine micro-particles
AA	Alopecia Areata
CaCl-Ca^2+^	Calcium chloride
PGE2	Prostaglandin E2
LIF	Leukemia-inhibiting factor
EBM	Evidence-based medicine
WKS	Weeks
MOS	Months.
b-FGF	Basic-Fibroblast Growth Factor
FGF-7	Fibroblast Growth Factor-7.
